# Gamification increases medical student confidence in surgical anatomy

**DOI:** 10.1111/medu.15627

**Published:** 2025-02-27

**Authors:** Reagan Lee, Luca Kovacs, Jingjing Wang, Beatrice Lofthouse, Katie Hughes

## 1 WHAT PROBLEM WAS ADDRESSED?

Between 2020 and 2022, UK medical students had reduced opportunities to learn anatomy due to COVID‐related cancellations of in‐person cadaveric anatomy dissection and/or prosection sessions. Consequently, students may have reduced confidence in anatomy knowledge, limiting participation and learning during surgical placements.

To address this issue, simulated ‘game‐style’ surgical anatomy tutorials were conducted to supplement undergraduate anatomy teaching by fifth‐year medical students for students beyond their fourth‐year of study at our local institution. Teaching was delivered from November 2023 to February 2024.

## 2 WHAT WAS TRIED?

An eight‐part synchronous online series was hosted on MedAll, an open‐access health care content website (https://app.medall.org/search?q=esss&search_on_demand_videos=true&boost_editors_pick=2). Each 90‐min session was themed around a surgical specialty, covering three interactive clinical cases. Each case was delivered by one fifth‐year medical student who presented key background knowledge before guiding students through an interactive ‘game‐style’ surgery. Cases were chosen based on common procedures and anatomy; a student would likely encounter on surgical placement and to reflect the undergraduate curriculum's intended learning outcomes (e.g. mediastinal anatomy in lobectomy). A ‘choose‐your‐own‐adventure’ format was used as the ‘game‐style’ component—students were involved in active decision‐making and anatomical structure identification through anonymous multiple‐choice group polling. This was performed at selected case junctures, illustrated by open‐access surgery videos and annotated diagrams. Each decision triggered unique consequences (and explanations); wrong answers result in quippy feedback from ‘the mean registrar’ whilst correct answers would elicit affirmation from ‘the nice consultant’. Sessions concluded with anonymous multiple‐choice questions with explanations for correct and incorrect answers.

Anonymous polls and feedback forms (5‐point Likert Scales, free‐text questions) assessed student experience.

Tutorial recordings were uploaded to MedAll. Subsequent asynchronous viewership was not analysed.

## 3 WHAT LESSONS WERE LEARNED?

Students reported increased confidence in anatomical and surgical knowledge after attending sessions. Composite scores from 168 students showed the mean self‐reported confidence in anatomical/surgical knowledge increased from 2.54 to 4.03 (5, *most confident*). Students reported enjoying the series' realism, interactivity and organisation with a composite score of 4.55 (5, *most relevant*). Feedback highlighted that gamifying surgical cases improved ‘interactivity’ and was ‘as if [students] were there themselves’.

This approach aided in simplifying complex anatomy through fun, case‐based activities to foster engagement. With the anonymous decision‐making format and low‐pressure environment of a peer‐led tutorial, students found the sessions a safe space to apply knowledge, while receiving high‐yield explanations for correct answers. This reinforced understanding of content while maximising learners' confidence.

Peer‐led teaching does have challenges. Variable teaching styles could reduce standardisation of content delivery and create an inconsistent learner experience.^1^ However, we found benefits in removing these traditional hierarchies. We felt it encouraged students to participate confidently without perceived judgement from senior colleagues. In future, we aim to mitigate the aforementioned issues through clearer standardisation guidelines and pilot sessions with tutors acting as attendees.

We hope this ‘game‐style’ approach to teach surgically relevant anatomy will be integrated into undergraduate medical education. This accessible technique could contribute to improving students' learning experiences while broadening their understanding of surgery in an increasingly online world.

## Data Availability

The data that support the findings of this study are available from the corresponding author upon reasonable request.
